# Rare case of ovarian Sertoli–Leydig cell tumor in an adolescent: a case report

**DOI:** 10.1097/MS9.0000000000003792

**Published:** 2025-09-01

**Authors:** Ayesha Junaid, Fizza Yousuf, Rabia Zafar, Javeria Taj, Hermann Yokolo

**Affiliations:** a Department of Medicine, Dow University of Health Sciences (DUHS), Karachi, Sindh, Pakistan; b Department of Gyne and Obs Unit 2, Civil Hospital Karachi, Karachi, Sindh, Pakistan; c Department of Research, Medical Research Circle (MedReC), Goma, Nord Kivu, DR Congo

**Keywords:** androgens, case report, Sertoli–Leydig cell tumor, sex cord stromal cells, virilization

## Abstract

**Introduction and Importance:**

Androgen-producing tumors are rare in females, typically arising from the ovaries or adrenal glands. Sertoli–Leydig cell tumors (SLCTs) are uncommon sex cord-stromal ovarian tumors that cause hyperandrogenism, leading to virilization symptoms such as hirsutism, deep voice, and amenorrhea. Their rarity and similarity with other androgen-secreting conditions make diagnosis challenging. This case report describes an SLCT in a 16-year-old girl, underscoring the value of early diagnosis and fertility-sparing surgery.

**Case Presentation:**

A 16-year-old girl presented with secondary amenorrhea, hirsutism, and voice deepening. Hormonal workup revealed elevated testosterone and low sex hormone-binding globulin. Imaging identified a solid mass in the left ovary. Adrenal causes were excluded via hormonal assays and imaging. The patient underwent a laparotomy with left salpingo-oophorectomy. Histopathology confirmed a moderately differentiated SLCT, and immunohistochemistry was positive for inhibin and calretinin. Postoperative CT showed no metastasis. She required no chemotherapy and remained under regular follow-up.

**Clinical Discussion:**

SLCTs represent less than 1% of ovarian tumors and primarily affect young women. They often present with signs of androgen excess. Diagnosis relies on clinical, hormonal, imaging, and histological data. DICER1 gene mutations are frequently associated. Surgical excision is the standard treatment, with fertility-sparing options preferred in early stages. Chemotherapy is reserved for advanced or poorly differentiated cases.

**Conclusion:**

SLCT is a rare but treatable ovarian tumor. Early detection of androgen excess is crucial. Surgery is effective, and fertility can be preserved. Larger registries are needed to enhance disease understanding and management strategies.

## Introduction

Androgen-producing tumors, while rare in women, can arise from various sources, with the ovaries and adrenal glands being the primary sites of origin. These tumors present a diagnostic challenge because of their rarity and the often-inconspicuous manifestations of androgen excess. Although ovarian tumors account for approximately 5% of all tumors in women, only about 1% of these are androgen-secreting tumors that cause hyperandrogenism^[1]^. Among this subset, Sertoli–Leydig cell tumors (SLCTs) represent a particularly rare form of sex cord-stromal tumors of the ovary, which are associated with a range of clinical presentations as a result of excess production of androgen. SLCTs being typically characterized by hyperandrogenism manifesting as virilization, hirsutism, deepening of the voice, clitoromegaly, and irregular or absent menstruation in affected women^[1,2]^. SLCTs can occur at any age, but approximately 75% of cases are diagnosed in women between the ages of 20 and 39. In contrast, less than 10% are seen in premenopausal or postmenopausal patients^[3]^. Hyperandrogenism is the excess production of androgens which are male hormones; however, they are also produced in females typically by ovaries and adrenal glands, but being in high amounts indicates an underlying pathological condition. Our aim is to report a unique case of a 16-year-old female with SLCT in the left ovary, and to analyze the clinical, diagnostic, and therapeutic characteristics of this rare tumor keeping in mind fertility sparing of this young patient. Being a rare tumor, this case report contributes to the limited body of literature on SLCTs, improving our understanding of the disease, ultimately leading to better diagnostic and treatment strategies in future research.

**HIGHLIGHTS**

- Rare ovarian SLCT diagnosed in a 16-year-old with signs of virilization.
- Hormonal and imaging findings guided diagnosis of androgen-secreting tumor.
- Histopathology confirmed SLCT with positive inhibin and calretinin markers.
- Fertility-sparing surgery was successfully performed with no chemotherapy.
- Multidisciplinary approach enabled early diagnosis and optimal management.

This case report has been reported in line with the SCARE checklist^[4]^.

## Case presentation

### Patient information

A 16-year-old unmarried young female presented to the gynecological outpatient department with secondary amenorrhea and signs of virilization. She had menarche at 14 years, with previously regular menstrual cycles lasting 4 days every 30 days for approximately 1.5 years. Nine months prior, she experienced sudden onset of amenorrhea, followed by hirsutism and voice deepening over the past 4 months. She denied pelvic pain, dysmenorrhea, menorrhagia, vaginal discharge, abdominal distension, or weight gain but reported a loss of appetite. Her past medical and surgical histories were unremarkable. She had no history of hormonal medication use or substance abuse. The patient was allergic to red meat and had no family history of gynecological cancers.

### Clinical findings

Initial diagnostic workup showed elevated testosterone (168 ng/dl), free androgen index (20), prolactin (42.80 ng/ml), thyroid stimulating hormone (8.110 IU/ml), decreased sex hormone-binding globulin (29.2 nmol/l), normal follicle-stimulating hormone (0.54 mIU/ml), and luteinizing hormone (6.97 mIU/ml). Levels of free T-4 (1.23 ng/dl), free T-3 (3.38 pg/ml), anti-thyroglobulin Abs (1.0 IU/mol), anti-thyroid peroxidase (Th, microsomal) (0.8 IU/mol), creatinine (0.7 mg/dl), and lactate dehydrogenase (200 U/L) were all in normal range. Alpha fetoprotein (25.80 IU/ml) was raised but the rest of tumor markers, including CA 19-9 (22.00 U/ml), CA-125 (11.50 U/ml), and beta-HCG (0.20 mIU/ml), were also normal. Baseline investigations showed no remarkable findings. All the laboratory findings are summarized in Table 1.

**Table 1:** Paraclinical assessments carried upon admission of the patient

Variables	Paraclinical assessments upon arrival	Normal values
Testosterone	168 ng/dl	6–58 ng/dl
Free androgen index	20	7–10
Prolactin	42.80 ng/ml	3.8–23 (non-pregnant)
Thyroid stimulating hormone	8.110 IU/ml	0.4–4.5 IU/ml
Sex hormone-binding globulin	29.2 nmol/l	34–148 nmol/l (premenopausal)
Follicle stimulating hormone	0.54 mIU/ml	1.4–9.9 mIU/ml (follicular phase)
Luteinizing hormone	6.97 mIU/ml	1.7–15 mIU/ml (follicular phase)
Free T-4^a^	1.23 ng/dl	0.8–2.7 ng/dl
Free T-3^b^	3.38 pg/ml	2.1–4.4 pg/ml
Anti-thyroglobulin abs	1.0 IU/mol	<4.1 IU/mol
Anti-TPO^c^ (th. microsomal)	0.8 IU/mol	<5.61 IU/mol
Creatinine	0.7 mg/dl	0.6–1.1 mg/dl (females)
Lactate dehydrogenase	200 U/L	<436 U/L (females)
Alpha fetoprotein	25.80 IU/ml	<8.2 IU/ml
CA^d^ 19-9	22.00 U/ml	<37 U/ml
CA-125	11.50 U/ml	<35 U/ml
Beta-HCG^e^	0.20 mIU/ml	<16 mIU/ml (non pregnant)

^a^ Free T-4, thyroxine.

^b^ Free T-3, triiodothyronine.

^c^ TPO, thyroid peroxidase.

^d^ CA, cancer antigen.

^e^ HCG, human chorionic gonadotropin.

Based on hyperandrogenism, signs of virilization, and secondary amenorrhea, differential diagnosis of androgen-secreting ovarian tumor and late onset congenital adrenal hyperplasia was made. On pelvic ultrasound, the left ovary was not visualized separately, showing solid mass of 6.0 × 4.1 cm suggesting left ovarian cyst. Non-contrast magnetic resonance imaging (MRI) of abdomen and pelvis revealed normal symmetric adrenal glands and left adnexal lesion of 5.5 × 5.9 × 4.9 cm (CC × TS × AP). Hence, androgen-secreting adrenal tumor and late onset adrenal hyperplasia were ruled out on the basis of normal serum 17-hydroxyprogesterone (0.47 ng/ml; normal range: 0.15–0.7 ng/ml in follicular phase), serum DHEA-SO4 – dehydroepiandrosterone sulfate (164.00 ug/dl; normal range 35–430 ug/dl), and normal adrenal glands on imaging. Based on these findings, a provisional diagnosis of androgen-producing ovarian tumor was made.

### Therapeutic intervention

**Figure 1.**
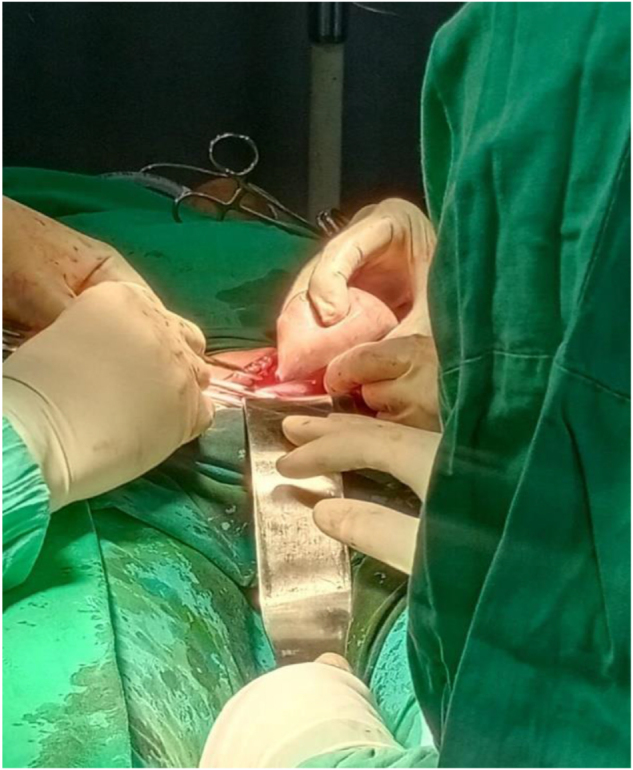
Intra-operative images showing left ovarian mass being resected.

**Figure 2.**
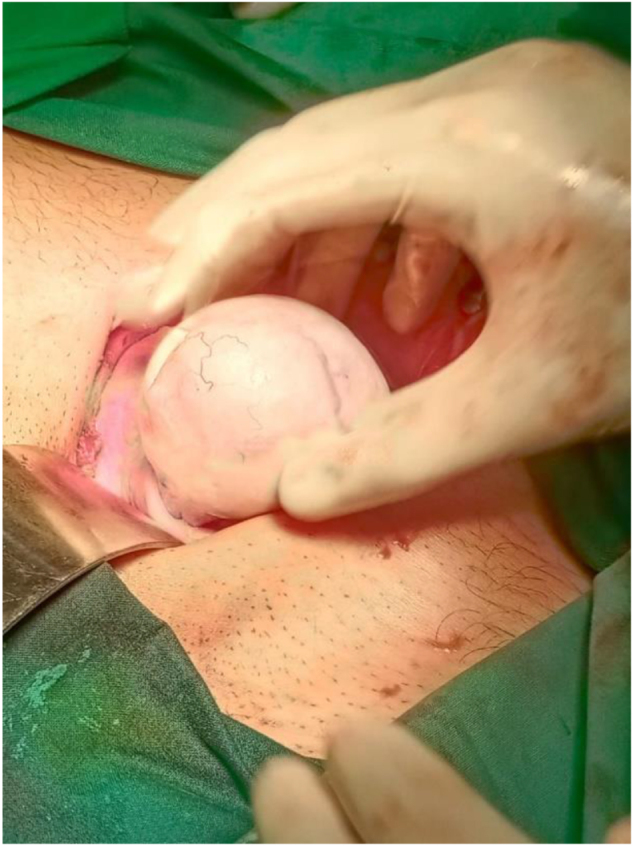
Intra-operative images showing left ovarian mass being resected.

**Figure 3.**
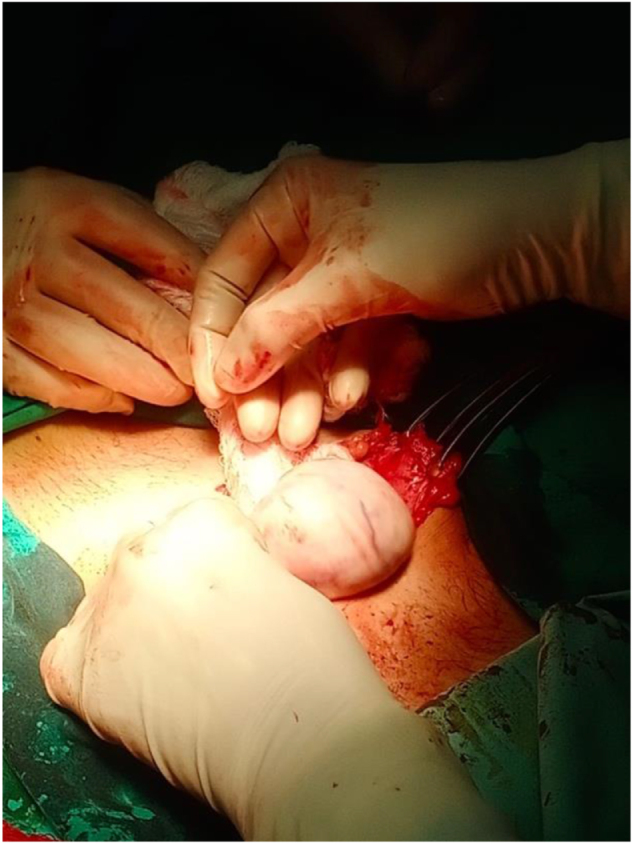
Intra-operative images showing left ovarian mass being resected.

The case was then discussed at the gynecology multidisciplinary team meeting and the decision was made to proceed with laparotomy and left salpingo-oophorectomy. Intraoperatively, a left ovarian cystic mass of 6 × 5 cm adherent to the left tube was observed with abnormal left ovarian tissue, as shown in Figures 1**–**3. The right ovary and tube were normal. The specimen was sent for histopathological examination (HPE) for confirmed diagnosis. Ascites were not present and further peritoneal washing was performed and sent for cytology to exclude malignancy.

### Follow-up and outcome

HPE of the left ovary revealed an intact nodular mass, measuring 5.5 × 5 × 4 cm, with a preserved capsule and an attached fallopian tube that measured (4 × 0.7 cm). On section, the tumor surface was variegated, dominated by a yellow-brown color with gray-brown hemorrhagic foci. Microscopically, the tumor showed hypo- and hypercellular areas. Approximately 80% consisted of cords of open and compressed primitive tubules, lined by columnar cells with oval to rounded nuclei and visible nucleoli. The remaining 20% contained cell clusters with rounded nuclei, abundant eosinophilic cytoplasm, spindle cells, and mitotic activity of two to three figures per high-power field. The fallopian tube showed no significant abnormalities. The initial differential diagnosis suggested hematopoietic stem cell lymphoma (HSCL) or juvenile granulosa cell tumor, but the morphological features and history favored HSCL. Immunohistochemistry confirmed a neoplastic proliferation with diffuse and lobulated growth, composed of Sertoli cells with mild to moderate atypia, forming sheets, tubules, and cords, accompanied by rare isolated Leydig cells. Calretinin and inhibin markers were positive, establishing the final diagnosis of moderately differentiated SLCT of the left ovary. Sarcomatous components and necrosis could not be proven. Post-operatively, computed tomography (CT) scan was performed which showed no significant hepatic, adrenal, pleuro-pulmonary, or osseous metastasis. After CT scan, in the opinion of expert doctors, it was decided that the patient does not require adjuvant chemotherapy rather will be kept on follow-up.

## Discussion

### Summary of results

This report describes a rare case of an ovarian SLCT in a 16-year-old adolescent girl presenting with secondary amenorrhea and signs of virilization. Hormonal evaluation revealed hyperandrogenism, and pelvic imaging identified a solid left ovarian mass. Surgical management with left salpingo-oophorectomy allowed for histopathological diagnosis, which was confirmed through immunohistochemical staining positive for inhibin and calretinin, consistent with a moderately differentiated SLCT. Postoperative follow-up was uneventful, and no adjuvant chemotherapy was indicated.

The conclusions are supported by the combination of clinical features (virilization), biochemical findings (elevated testosterone), imaging results (unilateral ovarian mass), and histological confirmation (Sertoli and Leydig cell components). This case highlights the importance of early recognition of hyperandrogenic signs, enabling prompt intervention and fertility-sparing management, with a favorable short-term prognosis.

### Relevant literature

SLCTs are rare ovarian sex cord-stromal neoplasms. They represent less than 0.5% of all ovarian tumors and approximately 1% of sex cord-stromal tumors^[3,5]^. Approximately 75% occur before the age of 30 years, most often in young women or adolescent girls^[6]^. Our patient, a 16-year-old girl with signs of hyperandrogenism, fits this profile. Clinically, SLCTs manifest as progressive virilization due to androgen secretion, including hirsutism, deep voice, acne, and amenorrhea^[1,2]^. Elevated serum testosterone associated with normal DHEA-SO4 levels, observed in our patient, suggests an ovarian origin of the androgen excess. Imaging revealed a solid unilateral adnexal mass, consistent with the typical features of sacrococcygeal teratoma (SCT) on ultrasound and MRI^[7]^.

Genetic studies have highlighted the role of DICER1 gene mutations in the pathogenesis of SLCT^[8,9]^. DICER1 encodes an enzyme essential for microRNA processing. Mutations can disrupt post-transcriptional regulation, thereby increasing susceptibility to several tumors, including SLCT^[10]^. Although germline testing was not performed in our case, it may be warranted in recurrent, bilateral, or familial settings. Treatment depends on age, fertility preferences, tumor differentiation, and disease stage. In young patients with early-stage disease, unilateral salpingo-oophorectomy is the preferred approach^[1,11]^. More extensive surgery is reserved for poorly differentiated or advanced tumors^[12]^. Our patient underwent fertility-sparing surgery without complications and with a favorable early recovery. Adjuvant chemotherapy is not routinely indicated for well- or moderately differentiated tumors, except in cases of poor prognosis^[13–15]^. When necessary, platinum-based regimens such as BEP (bleomycin, etoposide, and cisplatin) are used^[16,17]^. In this case, no chemotherapy was administered due to the absence of adverse effects.

To further our understanding, we reviewed recent reports from 2023 to 2025. Muscat and Calleja-Agius described a 14-year-old girl with similar clinical features^[3]^. Liu *et al* reported five cases of SLCT in adolescent girls, most of which were successfully treated with fertility-sparing surgery^[6]^. These results reinforce the importance of early screening and personalized management in young patients with androgen-secreting ovarian tumors.

### Future implications and take away lessons

This case highlights several important clinical lessons. In adolescent girls presenting with rapid virilization accompanied by secondary amenorrhea, tumor-related hyperandrogenism should be considered early. The combination of targeted hormonal evaluation and pelvic imaging allows prompt identification of an ovarian origin. A multidisciplinary approach involving gynecology, endocrinology, radiology, and pathology proved essential for accurate diagnosis and fertility-sparing surgical treatment. This experience encourages a more proactive diagnostic attitude toward unexplained hyperandrogenic signs in adolescents and a systematic consideration of sex cord-stromal tumors in the differential diagnosis. Increased vigilance can help reduce diagnostic delays and optimize patient management strategies.

### Strengths and limitations

This case report presents several important strengths. First, it highlights a rare ovarian neoplasm SLCT diagnosed at an early stage in an adolescent girl, allowing for fertility-preserving surgery with excellent prognosis. Moreover, it demonstrates strong multidisciplinary collaboration between gynecology, endocrinology, radiology, pathology, and surgery, which ensured a thorough and coordinated approach to diagnosis and treatment. Additionally, it contributes valuable insights to the limited literature on SLCTs and underscores the diagnostic value of combining hormonal assays, imaging, histopathological analysis, and immunohistochemistry. Nevertheless, this case also has some limitations. As a single observation, its findings cannot be generalized to all SLCT cases. Furthermore, genetic testing for DICER1 mutations, which could have provided deeper molecular insights, was not performed due to limited local resources. Finally, the lack of long-term follow-up data restricts our ability to assess recurrence or late outcomes. Despite these constraints, the case remains clinically relevant and supports the need for broader registries, genetic evaluation, and multidisciplinary collaboration in managing rare ovarian tumors.

## Conclusion

SLCT is a rare histologic subtype of ovarian SCSTs commonly presenting with symptoms of hyperandrogenism and amenorrhea. Identifying the source of androgens is crucial in early diagnosis and management. Surgery remains the primary treatment option, and fertility-sparing in young patients is achievable. This tumor has an excellent prognosis. The ideal chemotherapy has yet to be determined. For such rare cases, the establishment of regional, national, and worldwide registries should be prioritized to increase availability of comprehensive data, and improve our understanding of the disease, ultimately leading to better diagnostic and treatment strategies in subsequent research.

## Data Availability

Not applicable.
